# Effect of Structured Training on ICU Nurses' Knowledge‐Based Competence in Ventilator‐Associated Pneumonia Prevention in a Resource‐Limited Setting: An Explanatory Sequential Mixed‐Methods Study

**DOI:** 10.1002/nop2.70662

**Published:** 2026-06-23

**Authors:** Yakubu H. Yakubu, Muniru Mohammed Saani

**Affiliations:** ^1^ Goldfields University Department of Rural Health, Faculty of Health Sciences Curtin University Kalgoorlie Australia; ^2^ Faculty of Critical Care Nursing, Division of Nursing Ghana College of Nurses and Midwives Accra Ghana; ^3^ Department of Intensive Care Unit Tamale Teaching Hospital Tamale Ghana

**Keywords:** education, intensive care, mixed methods, nurse knowledge‐based competence, resource‐limited settings, ventilator‐associated pneumonia

## Abstract

**Aim:**

To evaluate the effect of a structured training intervention on ICU nurses' knowledge‐based competence in VAP prevention in a resource‐limited setting and identify personal, environmental and organisational barriers to implementation.

**Design:**

An explanatory sequential mixed‐methods design was employed, integrating a single‐group quasi‐experimental pre‐test–post‐test quantitative strand with a qualitative interview strand. Because participant responses were not linked across assessment points, findings represent group‐level improvements rather than within‐individual change.

**Methods:**

ICU nurses completed pre‐test (*n* = 57) and post‐test (*n* = 56) assessments using an adapted eight‐item VAP prevention knowledge questionnaire. A structured 3‐h workshop on VAP prevention bundles, infection control and airway management was delivered between tests; the post‐test occurred within 2 weeks. Group differences were analysed using an independent‐samples *t*‐test and chi‐square tests. Qualitative interview data (*n* = 8) were analysed thematically to explain and contextualise the quantitative findings.

**Results:**

Mean knowledge scores increased from 3.63 (SD 1.57) to 5.29 (SD 1.57) out of 8 (t(111) = −5.61, *p* < 0.001; *d* = 1.06). Because participant responses were not linked across assessment points, findings represent group‐level improvements rather than within‐individual change. The proportion classified as high knowledge‐based competence increased from 24.6% to 64.3% (*χ* ^2^(2) = 22.43, *p* < 0.001). Experience variables did not predict post‐test scores. Qualitative findings showed that shortages of supplies/equipment, unreliable power, absent protocols, limited supervision and high workload restricted consistent implementation. These findings highlight that improvements in knowledge may not translate into consistent bedside practice without system‐level support in resource‐limited ICUs.

**Conclusions:**

A brief, context‐specific training workshop significantly improved ICU nurses' knowledge‐based competence in VAP prevention. However, systemic barriers—equipment shortages, absent protocols, limited supervision and workload pressures—constrain the translation of knowledge gains into consistent bedside practice. Sustainable improvement requires embedding education within broader quality improvement efforts.

**No Patient or Public Contribution:**

No patient or public contribution.

## 1 Introduction

Healthcare‐associated infections (HAIs) remain a major global health concern, particularly within intensive care units (ICUs), where patients are critically ill, frequently exposed to invasive devices and often immunocompromised (Blot et al. 2022; Matthaiou et al. 2015; Odoom and Donkor 2025). Although ICUs constitute a small proportion of hospital beds, they account for a disproportionately high number of HAIs, exceeding 20% of all hospital‐acquired infections, thereby contributing significantly to increased morbidity, mortality and healthcare costs (Burillo and Bouza 2014; Tuma et al. 2023). Among these infections, ventilator‐associated pneumonia (VAP) stands out as the most prevalent and fatal, affecting between 10% and 40% of mechanically ventilated patients worldwide (Ferrer and Torres 2018; Piriyapatsom et al. 2016).

VAP is defined as pneumonia that develops 48 h or more after endotracheal intubation and mechanical ventilation. It is associated with prolonged ICU stays, heightened resource utilisation and increased mortality, particularly in low‐ and middle‐income countries (LMICs), where incidence rates can reach as high as 47.9 cases per 1000 ventilator days (Aloush and Al‐Rawajfa 2020; Kallet 2015; Nemet et al. 2025). Such regional variations are largely attributable to differences in infection prevention practices, adherence to evidence‐based guidelines, staffing ratios and the availability of essential equipment (Papazian et al. 2020). Compelling evidence indicates that the consistent implementation of VAP prevention bundles encompassing optimal patient positioning, suctioning protocols and ventilator circuit management can markedly reduce infection rates and improve patient outcomes (Martinez‐Reviejo et al. 2023; McNally et al. 2006; Singh and Abdullah 2024). However, in resource‐limited ICUs, implementation of these bundles remains inconsistent and understanding the interaction between nurse knowledge‐based competence and system‐level constraints is critical to improving patient outcomes.

In this study, knowledge‐based competence refers to the cognitive component of clinical competence, specifically nurses' knowledge and understanding of evidence‐based ventilator‐associated pneumonia prevention practices. Unlike comprehensive clinical competence, which also encompasses psychomotor skills, attitudes, clinical judgement and observed performance, knowledge‐based competence represents the extent to which nurses possess the theoretical knowledge required to guide safe and effective practice. Accordingly, the present study assessed the cognitive domain of competence using a structured knowledge questionnaire rather than direct observation of bedside performance.

## 2 Background/Justification for the Study

Despite the availability of these interventions, adherence remains suboptimal in many resource‐limited settings. In ICUs within tertiary referral settings in Ghana, systemic constraints including inadequate critical care training, limited human resources, non‐standardised protocols and insufficient equipment continue to undermine effective VAP prevention. ICU nurses, as the primary caregivers responsible for infection control, play a pivotal role in implementing preventive strategies. However, knowledge gaps and limited knowledge‐based competence frequently impede the translation of evidence‐based guidelines into routine practice (Kalyan et al. 2020; Madhuvu et al. 2020).

Previous studies in LMIC ICUs report persistent deficits in nurses' VAP‐related knowledge and practice (Bankanie et al. 2021; Gundo et al. 2021). While these studies documented knowledge deficits and educational needs, they relied predominantly on cross‐sectional surveys without intervention components, leaving key implementation questions unresolved: specifically, whether structured training can shift nurses from low to high competence categories and why system‐level barriers persist even when knowledge improves. The present study addresses both questions through an explanatory sequential mixed‐methods design. Yet, there is limited empirical evidence evaluating the impact of structured, context‐adapted training on ICU nurses' knowledge‐based competence. This study draws on Benner (1982)'s Novice‐to‐Expert Model and Bandura and Adams (1977)'s Self‐Efficacy Theory to examine how experiential learning and perceived confidence influence VAP‐prevention knowledge‐based competence in resource‐limited ICUs. This study provides one of the few explanatory sequential mixed‐methods evaluations of VAP‐prevention knowledge‐based competence in a resource‐limited ICU setting, offering both quantitative and contextual insights. These findings are particularly relevant for global nursing practice, especially in resource‐limited settings where system constraints significantly influence care delivery.

## 3 Aims and Objectives

This study aimed to evaluate ICU nurses' knowledge‐based competence in VAP prevention and examine factors influencing implementation.

The specific objectives were to:

- Assess ICU nurses' baseline knowledge‐based competence in VAP prevention.
- Evaluate the effect of structured training on post‐intervention knowledge‐based competence.
- Examine predictors of post‐training knowledge‐based competence.
- Explore personal, environmental and organisational barriers to VAP prevention implementation.

## 4 Design and Methods

### 4.1 Study Design

**FIGURE 1:**
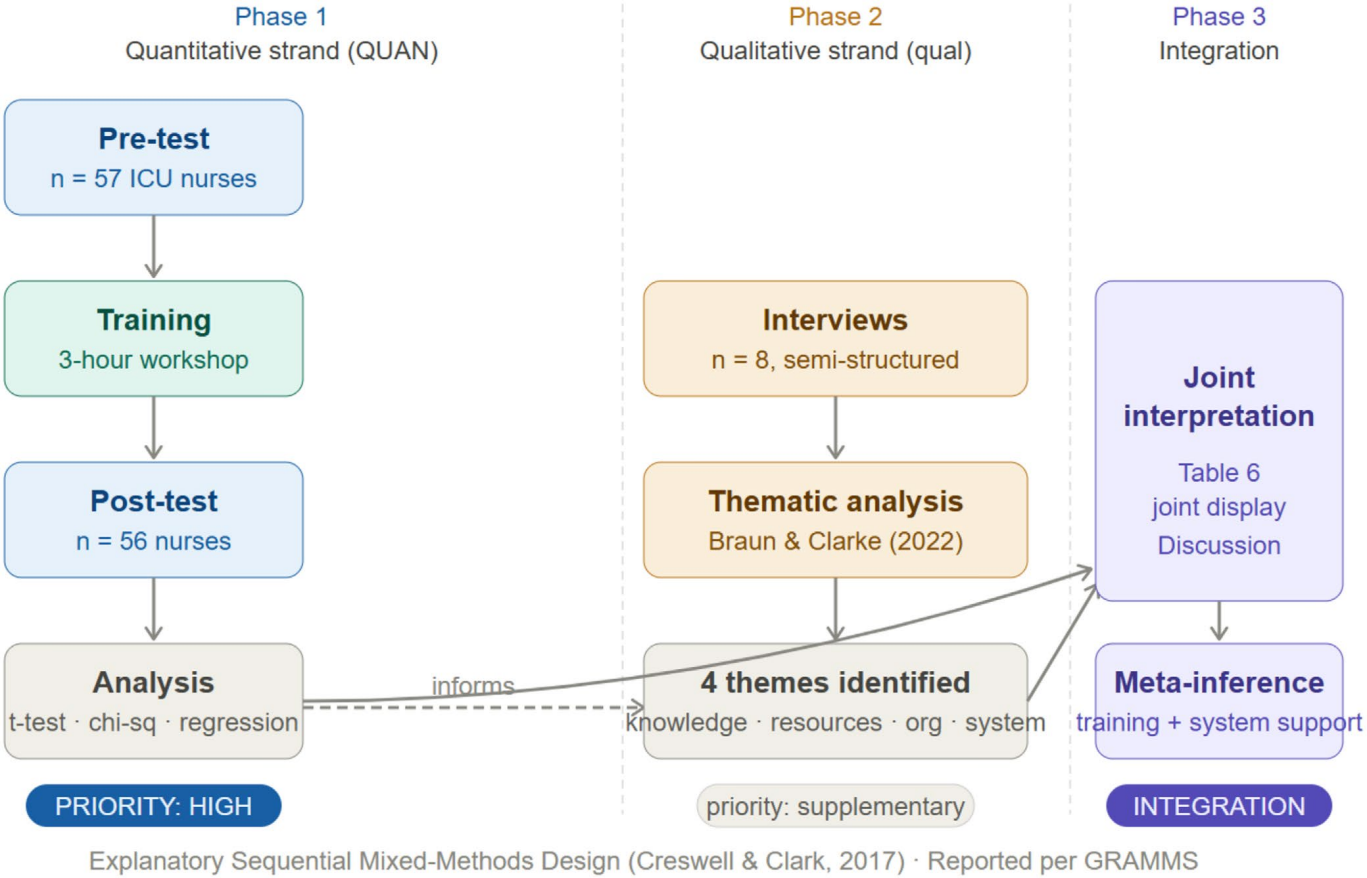
Explanatory sequential mixed‐methods design illustrating the sequence, priority and integration points of the quantitative and qualitative strands.

This study employed an explanatory sequential mixed‐methods design. Integration of the quantitative and qualitative strands occurred at the interpretation stage, where qualitative findings were used to explain and contextualise the quantitative results. The quantitative strand used a single‐group quasi‐experimental pre‐test–post‐test approach to evaluate the effect of a structured training intervention on ICU nurses' knowledge‐based competence in VAP prevention. The qualitative strand, comprising semi‐structured interviews with eight ICU nurses, was conducted after the post‐test phase to explain and contextualise the quantitative findings (Creswell and Clark 2017). The qualitative strand was informed by quantitative findings showing improvement in knowledge‐based competence alongside persistent moderate scores among some participants. Interview questions therefore focused on explaining how nurses applied VAP prevention knowledge in practice and what individual, organisational and system‐level factors limited implementation. Figure 1. Visual representation of the explanatory sequential mixed‐methods design, illustrating the sequence, priority and integration point of the quantitative and qualitative strands. QUAN = quantitative strand (high priority); qual = qualitative strand (Supporting Information). Integration occurred at the interpretation stage through a joint display (Table 6) and synthesis in the Discussion. Reporting of this study followed the Good Reporting of A Mixed Methods Study (GRAMMS) recommendations, the Consolidated Criteria for Reporting Qualitative Research (COREQ) and the Transparent Reporting of Evaluations with Nonrandomized Designs (TREND) guidelines. Corresponding checklists are provided in the Supporting Information (Table S2 and Supporting Information Files S4 and S5).

### 4.2 Setting and Sample

The study was conducted in a tertiary teaching hospital, the principal referral centre in a resource‐limited setting. Data were collected from two intensive care units: the General Intensive Care Unit (GICU), a four‐bed unit staffed by 37 personnel and the Maternity Intensive Care Unit (MICU), a six‐bed unit staffed by 27 personnel. These units provide care for critically ill adult and obstetric patients requiring mechanical ventilation and advanced monitoring. The target population comprised all registered nurses actively employed in the GICU and MICU of the study setting, a large referral hospital, totalling 64. Participants were recruited through convenience sampling, selecting all eligible nurses present and on duty during the data collection period (Galloway 2005). This approach is acknowledged as a study limitation. Given the small and geographically concentrated target population of 64 ICU nurses across two units, recruiting all eligible available nurses was the most practical strategy to maximise participation and minimise selection bias within the accessible population. Inclusion criteria required registered nurses with a minimum of 3 months of active bedside experience. Nurses in managerial roles and those on clinical orientation were excluded. The Yamane (1973) formula was applied using a precision level of 0.05 and a 95% confidence interval, yielding a minimum required sample of 55 participants. The achieved sample of 57 pre‐test and 56 post‐test participants exceeded this threshold.

For the qualitative strand, purposive sampling was used to recruit information‐rich participants (Ahmad and Wilkins 2025; Palinkas et al. 2015). This approach ensured inclusion of participants capable of providing rich and relevant perspectives on VAP prevention practices.

### 4.3 Data Collection Tools and Methods

#### 4.3.1 Training Intervention

Between the pre‐test and post‐test assessments, participants attended a structured training workshop on VAP prevention delivered by the principal investigator. The training content was organised into four thematic modules: (1) the definition, aetiology and pathophysiology of VAP; (2) evidence‐based VAP prevention bundles, including patient positioning, oral hygiene and ventilator circuit management; (3) airway management and suctioning techniques; and (4) infection control principles specific to the ICU setting. Content was delivered using projected PowerPoint presentations prepared from peer‐reviewed literature and current clinical guidelines. The training was conducted as a single group workshop session of approximately 3 hours. To ensure content validity, materials were reviewed by two independent intensivists and by the Deputy Director of Nursing Services and the GICU Nurse Manager of the study setting, both critical care specialists. A post‐test was administered within 2 weeks of the training workshop. All participants received identical educational content delivered by the principal investigator using the same PowerPoint materials, teaching sequence and duration. Training content was validated before delivery to ensure consistency and fidelity. Attendance was voluntary; all eligible ICU nurses present and on duty during the scheduled workshop date were invited to participate and all attendees received identical educational content.

The workshop incorporated didactic instruction, group discussion and question‐and‐answer sessions. The format was primarily didactic; hands‐on clinical skills practice and case‐based simulation were not included, which is acknowledged as a limitation.

#### 4.3.2 Data Collection Instrument

Demographic data were collected using a nine‐item biographic questionnaire. Knowledge‐based competence in VAP prevention was assessed using an adapted version of the evidence‐based VAP prevention questionnaire developed by Labeau et al. (2007), comprising nine multiple‐choice items. Item 8, addressing the advantage of kinetic beds, was removed because kinetic beds are not available at the study setting. The adapted instrument therefore comprised eight items, with total scores ranging from 0 to 8. Scores were converted to percentages and categorised into three knowledge‐based competence levels: low (0%–37%), moderate (38%–62%) and high (63%–100%), consistent with cut‐offs applied in comparable studies (Bankanie et al. 2021; Blot et al. 2007). Following removal of the kinetic‐bed item, the adapted questionnaire underwent face and content review by two critical care specialists and the Deputy Director of Nursing Services to ensure contextual relevance and alignment with evidence‐based VAP prevention practices within the study setting. The instrument was also piloted among five ICU nurses outside the study sample to assess clarity and comprehension and no further modifications were required. An open‐ended supplementary question elicited perceived barriers to VAP prevention across personal, environmental and organisational domains.

Internal consistency reliability of the adapted eight‐item instrument was evaluated using the Kuder–Richardson Formula 20 (KR‐20) (Kuder and Richardson 1937). KR‐20 coefficients were 0.32 for the pre‐test sample and 0.42 for the post‐test sample (Bankanie et al. 2021; Labeau et al. 2007).

The interview guide was developed based on the study objectives and informed by Benner's Novice‐to‐Expert Model and Bandura's Self‐Efficacy Theory, ensuring exploration of competence development, experiential learning and contextual barriers to VAP prevention (see Supporting File S1: Interview guide).

#### 4.3.3 Data Collection Procedure

Pre‐test questionnaires were distributed during a scheduled staff meeting. Post‐test questionnaires were administered within 2 weeks of the training workshop. One participant did not complete the post‐test (*n* = 56; response rate 87.5%); the reason for non‐completion was not recorded. Semi‐structured interviews were conducted individually with eight purposively selected ICU nurses following post‐test completion until thematic saturation was reached (Saunders et al. 2018). Eight participants were purposively selected to reflect variation in ICU type, years of experience and involvement in the training intervention. Interviews continued until no substantially new codes or themes were identified in successive interviews. Saturation was assessed through ongoing review of interview summaries, coding notes and emerging themes. By the seventh and eighth interviews, responses repeated previously identified patterns relating to routine practice, resource constraints, protocols, supervision and workload, indicating thematic saturation.

### 4.4 Data Analysis

Data were analysed using IBM SPSS Statistics version 31. Descriptive statistics were computed to summarise participant characteristics and item‐level knowledge scores. Normality of knowledge scores was assessed using the Kolmogorov–Smirnov and Shapiro–Wilk tests, supplemented by skewness and kurtosis values. Both tests indicated significant deviation from normality (pre‐test: *p* = 0.010; post‐test: *p* = 0.008). However, skewness (0.317 and −0.026) and kurtosis (−0.387 and −0.999) values were within acceptable bounds (±1) and visual inspection supported approximate normality. Given the adequate sample sizes (*n* = 57 and *n* = 56) and equal variances confirmed by Levene's test (*F* = 0.18, *p* = 0.670), an independent‐samples *t*‐test was used to compare pre‐test and post‐test knowledge scores, which is appropriate for this between‐groups data structure.

Although the training was delivered to one underlying staff cohort, participant responses were not linked across assessment points; therefore, pre‐test and post‐test scores were analysed as independent groups. This limits inference about within‐person change. Cohen's *d* was calculated using the pooled standard deviation to quantify the practical magnitude of the training effect. Knowledge‐based competence level distributions were compared using a chi‐square test of independence. Multiple linear regression assessed the influence of years of RN experience, duration of ICU service and ICU type on post‐training scores. All tests were two‐tailed with *α* = 0.05. This study was reported in accordance with the GRAMMS framework for mixed‐methods studies (O'Cathain et al. 2008) (see Supporting File S2), the TREND checklist for non‐randomised evaluations (Des Jarlais et al. 2004) (see Supporting File S4) and the COREQ checklist for qualitative reporting (Tong et al. 2007) (see Supporting File S5).

Qualitative data were analysed using Braun and Clarke (2022) thematic analysis following their six‐phase framework. Audio recordings were transcribed verbatim and cross‐checked for accuracy. To enhance rigour, the first and second authors independently coded the data and discrepancies were resolved through discussion until consensus was reached.

### 4.5 Ethics Considerations

Ethical clearance was obtained from the Navrongo Health Research Centre Institutional Review Board (NHRCIRB519, approval date: 15th May 2023). Institutional permission was granted by the Tamale Teaching Hospital Chief Executive Officer (TTH/R&D/SR/147, dated 8th June 2023). All participants provided written informed consent. Participation was voluntary and confidentiality was maintained throughout. This study was conducted in accordance with the Declaration of Helsinki.

### 4.6 Rigour and Reflexivity

To ensure the trustworthiness of the qualitative findings, rigour was established through four criteria adapted from Lincoln and Guba (1986) framework: credibility, transferability, dependability and confirmability.

Credibility was strengthened through prolonged engagement with the data, member checking with two participants who confirmed that the emergent themes accurately reflected their experiences and investigator triangulation, whereby a second researcher independently coded a subset of three transcripts and the resulting themes were compared and reconciled through discussion. Transferability was supported by providing a thick description of the study context, participant characteristics and setting, enabling readers to assess the applicability of findings to similar resource‐limited ICU environments. Dependability was addressed through the maintenance of an audit trail, including reflective memos documenting analytical decisions made throughout the coding process. Confirmability was ensured by grounding all themes directly in participant data, with representative verbatim quotations provided to illustrate each theme and allow readers to evaluate the interpretive process independently.

Reflexivity was maintained throughout the study. The principal investigator, who conducted all interviews, was also involved in the quantitative arm of the study and was known to participants as a clinical supervisor within the institution. This dual role as both researcher and clinical peer carried the potential to introduce social desirability bias, whereby participants might frame their responses more favourably in the presence of a known supervisor. To mitigate this, participants were explicitly assured of the confidentiality of their responses before each interview, reminded that their answers would have no bearing on their employment or clinical standing and encouraged to speak freely about challenges and limitations in practice. Reflective memos were maintained after each interview to document the researcher's awareness of these dynamics and their potential influence on data interpretation.

### 4.7 Theoretical Framework

Benner (1982) Novice‐to‐Expert Model informed the study's focus on knowledge‐based competence as a developmental construct, underpinning the expectation that nurses with fewer years of ICU experience would demonstrate lower baseline VAP prevention knowledge and explaining why a single training episode may be insufficient for all nurses to achieve high knowledge‐based competence. Bandura's Self‐Efficacy Theory (Bandura and Adams 1977) informed the examination of barriers and the assumption that structured training, by building knowledge and confidence, can increase self‐efficacy for VAP prevention behaviours. Both frameworks are revisited in the Discussion.

### 4.8 Trial Registration

Not applicable to this study. This study was not registered as a clinical trial because it evaluated a nursing education intervention rather than a therapeutic or clinical treatment and did not involve patient participants.

### 4.9 Integration of Methods

Integration occurred at the interpretation stage (Creswell and Clark 2017; Wisdom and Creswell 2013). Quantitative findings identified significant improvements in knowledge‐based competence following training but could not explain why implementation challenges persisted. The qualitative strand was therefore used to explore contextual, organisational and environmental factors influencing the translation of knowledge into practice. Integration enabled explanation of the gap between improved post‐test scores and continuing barriers to VAP prevention implementation.

## 5 Results/Findings

### 5.1 Quantitative Results

#### 5.1.1 Study Participant Characteristics

A total of 57 participants completed the pre‐test (response rate: 89%) and 56 (response rate: 87.5%) completed the post‐test. The overall mean age was 31.6 years (SD = 4.2), ranging from 21 to 42 years. The majority were registered general nurses (84.1%), with very few having postgraduate training in intensive care nursing (1.8%). More than three‐quarters (77.9%) had between 1 and 5 years of ICU experience, with the majority working in GICUs (60.2%). Table 1 contains the study demographic data.

**TABLE 1:** Demographic characteristics of the study population.

Variable	Pre‐test (*n* = 57)	Post‐test (*n* = 56)	Total (*N* = 113)
Gender			
Male	37 (64.9%)	37 (66.1%)	74 (65.5%)
Female	20 (35.1%)	19 (33.9%)	39 (34.5%)
Age (years)			
21–25	4 (7.0%)	4 (7.1%)	8 (7.1%)
26–30	21 (36.8%)	18 (32.1%)	39 (34.5%)
31–36	19 (33.3%)	21 (37.5%)	40 (35.4%)
≥ 36	13 (22.8%)	13 (23.2%)	26 (23.0%)
Postgraduate training in ICU nursing			
Yes	1 (1.8%)	1 (1.8%)	2 (1.8%)
No	56 (98.2%)	55 (98.2%)	111 (98.2%)
Professional background			
Registered General Nurse (RGN)	48 (84.2%)	47 (83.9%)	95 (84.1%)
Critical Care Nurse (CCN)	1 (1.8%)	1 (1.8%)	2 (1.8%)
Other	8 (14.0%)	8 (14.3%)	16 (14.2%)
Years of experience as RN			
1–5	36 (63.2%)	36 (64.3%)	72 (63.7%)
6–10	18 (31.6%)	18 (32.1%)	36 (31.9%)
11–15	3 (5.3%)	2 (3.6%)	5 (4.4%)
Years of experience as ICU RN			
1–5	44 (77.2%)	44 (78.6%)	88 (77.9%)
6–10	10 (17.5%)	10 (17.9%)	20 (17.7%)
11–15	3 (5.3%)	2 (3.6%)	5 (4.4%)
Type of ICU			
General ICU (GICU)	34 (59.6%)	34 (60.7%)	68 (60.2%)
Maternity ICU (MICU)	23 (40.4%)	22 (39.3%)	45 (39.8%)

### 5.2 Reliability of the Adapted Instrument

KR‐20 coefficients for the modified eight‐item questionnaire were 0.32 (pre‐test) and 0.42 (post‐test), indicating low internal consistency. Given that the instrument evaluates multiple evidence‐based components of VAP prevention, the composite score should be interpreted as a broad knowledge index rather than a unidimensional psychometric scale.

### 5.3 Knowledge Scores and Training Effect

Table 2 presents the number of correct responses for each knowledge domain before and after the educational intervention. Improvements were observed across all domains, with the greatest increases occurring in frequency of humidifier changes (+17), open versus closed suction systems (+16), patient positioning (+16) and endotracheal tubes with subglottic secretion drainage (+13). The training intervention appeared particularly effective in addressing areas with lower baseline knowledge.

**TABLE 2:** Total score of correct answers for individual items.

Knowledge domain	Pre‐test (*n* = 57)	Post‐test (*n* = 56)	Δ
Oral vs. nasal route for endotracheal intubation	44	53	+9
Frequency of ventilator circuit changes	24	28	+4
Type of airway humidifier	28	34	+6
Frequency of humidifier changes	8	25	+17
Open vs. closed suction systems	22	38	+16
Frequency of change in suction systems	18	26	+8
ETT with subglottic secretion drainage lumen	28	41	+13
Patient positioning	35	51	+16
Total	207	296	+89

The mean pre‐test knowledge score was 3.63 ± 1.57, whereas the mean post‐test knowledge score increased to 5.29 ± 1.57 (Table 3). Independent‐samples *t*‐test analysis demonstrated a statistically significant group‐level improvement following the educational intervention (t(111) = −5.61, *p* < 0.001), with a mean difference of −1.65 (95% CI: −2.24 to −1.07) and a large effect size (Cohen's *d* = 1.06). The Mann–Whitney U test yielded a consistent result (*U* = 748.5, *p* < 0.001), supporting the robustness of the findings.

**TABLE 3:** Comparison of knowledge scores between pre‐ and post‐test groups.

Group	*N*	Mean (SD)	MD	95% CI	t(df)	*p*
Pre‐test	57	3.63 (1.57)	−1.65	−2.24 to −1.07	−5.61 (111)	< 0.001
Post‐test	56	5.29 (1.57)				

*Note:* Cohen's *d* = 1.06 (large effect). Levene's test: *F* = 0.18, *p* = 0.670 (equal variances assumed). Mann–Whitney *U* = 748.5, *p* < 0.001 (non‐parametric parallel).

#### 5.3.1 Changes in Knowledge‐Based Competence Categories

Nurses' knowledge‐based competence in VAP prevention was categorised into low, moderate and high levels (Table 4). At pre‐test, the majority were classified as low knowledge‐based competence (13/57, 22.8%) or moderate knowledge‐based competence (30/57, 52.6%), with 14 (24.6%) reaching the high level. Following training, knowledge‐based competence distributions shifted considerably: 1 out of 56 (1.8%) remained in the low category, 19 (33.9%) were in the moderate category and 36 (64.3%) were classified as high knowledge‐based competence.

**TABLE 4:** Knowledge‐based competence categories derived from VAP knowledge scores.

Knowledge‐based competence level	Pre‐test (*n* = 57)	Post‐test (*n* = 56)
Low knowledge‐based competence (0%–37%)	13 (22.8%)	1 (1.8%)
Moderate knowledge‐based competence (38%–62%)	30 (52.6%)	19 (33.9%)
High knowledge‐based competence (63%–100%)	14 (24.6%)	36 (64.3%)
Total	57 (100%)	56 (100%)

*Note:* Competence levels are based on percentage scores converted from the 0–8 raw score range: Low = 0%–37% (scores 0–2); Moderate = 38%–62% (scores 3–4); High = 63%–100% (scores 5–8). Cut‐offs are consistent with those applied by Labeau et al. (2007) and Bankanie et al. (2021).

A chi‐square test revealed a statistically significant association between group (pre vs. post) and knowledge‐based competence level, *χ* ^2^ (2, *N* = 113) = 22.43, *p* < 0.001 (Table 5). This suggests that training was associated with a shift from low and moderate knowledge‐based competence towards higher levels.

**TABLE 5:** Chi‐square test of independence: group × knowledge‐based competence level.

Test	Value	df	*p* (2‐sided)
Pearson chi‐square	22.428	2	< 0.001
Likelihood ratio	25.643	2	< 0.001
Linear‐by‐linear association	22.067	1	< 0.001

#### 5.3.2 Predictors of Post‐Training Knowledge‐Based Competence

A multiple linear regression analysis was performed to assess the influence of years of experience as a registered nurse (RN), duration of ICU service and ICU type (GICU vs. MICU) on ICU nurses' post‐training scores (*n* = 56). The overall model was not statistically significant (*F*(3, 52) = 0.94, *p* = 0.428), accounting for only 5.2% of the variance in post‐training scores (*R* ^2^ = 0.052, Adjusted *R* ^2^ = −0.003). None of the individual predictors significantly predicted post‐test knowledge‐based competence: years of RN experience (B = 0.117, *β* = 0.214, *p* = 0.285), duration in ICU (B = 0.011, *β* = 0.017, *p* = 0.932) and ICU type (B = 0.036, *β* = 0.011, *p* = 0.935). Collinearity diagnostics indicated no multicollinearity issues (VIF range: 1.008–2.143). The Durbin–Watson statistic (*d* = 2.18) confirmed independence of residuals.

### 5.4 Barriers to VAP Prevention

Open‐ended survey responses provided preliminary insights into barriers to VAP prevention. These findings were subsequently explored in greater depth through qualitative interviews. The following section presents participants' views on barriers to VAP prevention, grouped into three themes from the quantitative open‐ended responses.

Personal factors: Most participants identified limited staff knowledge as a significant barrier, with many lacking the technical expertise, ICU training and skills needed to prevent VAP. An unprofessional attitude, ineffective supervision and low job motivation further exacerbated these challenges.

Environmental factors: Participants cited unstable power supplies, shortages of suction catheters, face masks and ventilator accessories and the reuse of suction catheters across patients. Procurement challenges resulted in infrequent circuit changes and an inability to obtain closed‐system suction catheters. ‘we reused suction catheters several times on different patients after rewashing’.

Organisational factors: Inadequate staffing, lack of trained personnel, limited access to PPE, absence of in‐service training for staff caring for mechanically ventilated patients and lack of evidence‐based VAP prevention protocols were the primary systemic barriers identified.

To further explain the quantitative findings, particularly the observed improvements in knowledge alongside persistent implementation barriers, qualitative interview data were analysed thematically. Themes and subthemes are provided in Supporting File S3. The qualitative findings are presented below to add explanatory depth to the quantitative results.

## 6 Qualitative Findings

### 6.1 Practical Knowledge and Routine VAP Prevention Practices

This theme captures how ICU nurses conceptualise and operationalise VAP prevention within everyday clinical practice. Findings suggest that practice is largely task‐oriented and pragmatically driven, with emphasis placed on interventions that are feasible within existing constraints rather than comprehensive adherence to evidence‐based bundles. This theme helps explain the quantitative improvement in knowledge scores by showing that nurses already possessed routine experiential practices that were reinforced through training.

#### 6.1.1 Emphasis on Basic, Feasible Preventive Practices

Participants described VAP prevention primarily in terms of routine, task‐based interventions embedded in daily care. These included mouth care, suctioning, changing endotracheal tube strapping and maintaining ventilator circuits. Among these, mouth care was consistently prioritised due to its perceived effectiveness and ease of implementation:

One of it is doing mouth care, routine mouth care for the patients… we have made it a habit in our ward to do mouth care for all the patients when we come for duty, daily. (P1)

This emphasis reflects an adaptive approach in which nurses prioritise interventions that are controllable and consistently achievable, even in the face of broader systemic limitations. However, this also suggests that VAP prevention may be reduced to isolated tasks rather than an integrated bundle approach, potentially limiting overall effectiveness.

#### 6.1.2 Infection Prevention Knowledge Acquired Through Experience

Participants indicated that their knowledge of VAP prevention was largely derived from clinical experience and self‐directed learning, rather than structured institutional training:

What has helped is my experience in the ICU… I've been in the ICU for quite a long time. (P3)

This reliance on experiential learning highlights a system where knowledge‐based competence development is individualised rather than standardised. While experience contributes to familiarity with practices, the absence of structured educational reinforcement may lead to variability in knowledge application and limit progression to higher levels of clinical expertise. Participants also described limited access to formal learning opportunities within the unit, reinforcing reliance on informal knowledge acquisition. This pattern of experience‐based, individually variable knowledge acquisition is consistent with the moderate baseline knowledge‐based competence scores observed quantitatively (pre‐test M = 3.63, SD = 1.57) and helps explain why structured training produced such a substantial effect filling gaps that experience alone had not addressed.

### 6.2 Resource and Infrastructure Constraints

This theme reflects how material and infrastructural limitations shape and constrain VAP prevention practices, often forcing nurses to adopt compensatory or improvised strategies. Although knowledge improved quantitatively, shortages of equipment and infrastructure limited implementation.

#### 6.2.1 Limited Availability of Equipment and Consumables

Participants consistently reported shortages of essential materials, including suction catheters and personal protective equipment, which directly affected adherence to infection prevention protocols:

You are looking for suction catheters… and you don't have the suction catheters because it is limited in the facility. (P5)

Such constraints often necessitated deviations from best practice, with nurses forced to prioritise immediate patient needs over ideal infection control standards. This highlights a critical gap between evidence‐based recommendations and the realities of care delivery in resource‐limited settings.

#### 6.2.2 Unstable Power Supply and System‐Level Failures

Unreliable electricity supply emerged as a major systemic barrier disrupting routine ICU care:

You can even try to suction a patient then all of a sudden there's no light. (P6)

These interruptions not only compromise timely clinical interventions but also increase patient vulnerability to complications such as VAP. This finding underscores the extent to which macro‐level infrastructural issues directly influence micro‐level clinical practice. These resource and infrastructure deficits provide a key explanation for why, despite the significant post‐training improvement in knowledge scores, a third of nurses (33.9%) remained in the moderate knowledge‐based competence category; the knowledge to act was present, but the conditions to act consistently were not.

### 6.3 Organisational and Training‐Related Barriers

This theme highlights how institutional structures and organisational culture influence the development and sustainability of VAP prevention knowledge‐based competence. These findings explain why improved knowledge‐based competence did not automatically translate into standardised practice.

#### 6.3.1 Absence of Standardised VAP Prevention Protocols

Participants described a lack of formalised, evidence‐based guidelines governing VAP prevention practices:

We don't have standard protocols for preventing VAP. We always rely on individual knowledge on prevention. (P2)

In the absence of standardised protocols, practice becomes highly individualised, leading to inconsistencies in care delivery and reduced accountability. This suggests a system where knowledge‐based competence is not institutionally reinforced, limiting the standardisation of best practices.

#### 6.3.2 Low Engagement in In‐Service Training

In‐service training was perceived as inadequate and undervalued within the organisational culture:

In‐service training… we have not taken it seriously over time. The workers' attitude is also another issue… they think that that person is trying to waste their time. (P4)

Low engagement was attributed to a combination of workload pressures, negative staff attitudes and organisational limitations. This reflects a weak culture of continuous professional development, which undermines the consolidation and sustainability of knowledge‐based competence gained through formal training interventions. The absence of protocols and a weak training culture explains why years of RN experience and ICU duration did not predict post‐test knowledge‐based competence in the regression analysis without standardised reinforcement, accumulated experience does not reliably translate into higher knowledge‐based performance.

### 6.4 Supervision, Workload and Systemic Support Challenges

This theme captures broader systemic and leadership‐related factors that influence the implementation of VAP prevention practices. System pressures contextualised the gap between educational gains and bedside implementation.

#### 6.4.1 Inadequate Clinical Supervision and Feedback

Participants reported limited supervisory oversight and absence of accountability mechanisms:

Those who are meant to supervise… most of the time does not even come around to see what you are doing. The system is open for anyone to do what he or she thinks is the best. (P7)

This lack of supervision reduces opportunities for feedback, reinforcement and correction of practice, thereby limiting the development of consistent and evidence‐based care.

#### 6.4.2 High Workload and Staffing Pressures

Heavy workloads and insufficient staffing were identified as key barriers to consistent VAP prevention:

Because of the lack of numbers, the other patients will be left to their own pace. (P8)

Under such conditions, nurses are forced to prioritise urgent clinical tasks, often at the expense of preventive care. This results in a shift from preventive to reactive practice, undermining infection control efforts.

#### 6.4.3 Delayed Diagnostic Feedback and Family Financial Constraints

System inefficiencies and socio‐economic factors further complicated patient care:

You never get the report for the swab they have done. (P2)

After one week it becomes very difficult for them to secure medications. (P5)

Delayed laboratory feedback limits timely clinical decision‐making, while financial constraints affecting families disrupt continuity of care, particularly in relation to nutrition and medication. These findings highlight how health system limitations and socio‐economic realities intersect to influence clinical outcomes. Taken together, these systemic challenges illuminate the implementation gap between the group‐level knowledge gains demonstrated quantitatively and the persistent barriers to consistent VAP prevention practice reported in these interviews.

### 6.5 Integration of Quantitative and Qualitative Findings

The explanatory sequential mixed‐methods design enabled integration of quantitative and qualitative findings to provide a more comprehensive understanding of ICU nurses' knowledge‐based competence in VAP prevention. While the quantitative findings demonstrated significant improvements following training, the qualitative findings explained the contextual and organisational factors that influenced the translation of knowledge into practice. Table 6 presents the integrated meta‐inferences derived from both strands.

**TABLE 6:** Joint display integrating quantitative and qualitative findings.

Quantitative finding	Qualitative theme	Integration/meta‐inference	Practice implication
Knowledge score improved from 3.63 to 5.29	Practical knowledge and routine VAP prevention practices	Training reinforced existing experiential practices and filled knowledge gaps	Continue structured VAP education
High knowledge‐based competence increased from 24.6% to 64.3%	Organisational and training‐related barriers	Knowledge improved, but lack of protocols limited standardised practice	Introduce VAP checklist/protocol
33.9% remained moderate post‐test	Resource and infrastructure constraints	Knowledge alone was insufficient when supplies/electricity were unreliable	Strengthen supply chain and equipment availability
Experience variables did not predict post‐test scores	Supervision, workload and system support	Experience alone may not improve knowledge‐based competence without feedback and supervision	Implement mentoring, audit‐feedback and supportive supervision

## 7 Discussion

This mixed‐methods study examined whether a brief, structured workshop could improve ICU nurses' knowledge‐based competence in ventilator‐associated pneumonia (VAP) prevention in a resource‐limited setting and explored barriers to applying evidence‐based practices. The findings show a large improvement in knowledge‐based competence after training, but also a clear implementation gap: nurses reported persistent system constraints that limited consistent delivery of recommended VAP‐prevention bundles (Hellyer et al. 2016; Rehmani et al. 2024; Rosenthal et al. 2025). For clinical nursing practice, the results suggest that education is necessary but insufficient unless supported by protocols, supervision and basic supplies. The qualitative findings provided explanatory depth to the quantitative results, enabling a more comprehensive understanding of how knowledge gains were influenced by contextual and organisational factors. Participants were predominantly young registered general nurses with limited postgraduate ICU preparation, a pattern reported in other LMIC critical care contexts (Bankanie et al. 2021; Hopkinson et al. 2022; Osman et al. 2021; Saghafi et al. 2026). This aligns directly with the qualitative finding in Theme 1, where nurses attributed their VAP prevention practices to accumulated ICU experience rather than formal training—a pattern consistent with Benner's characterisation of competence as experientially rather than didactically acquired. Similarly, the supervisory and accountability gaps described in Theme 4 help explain why self‐efficacy for VAP prevention behaviours, as theorised by Bandura, remains insufficient to sustain consistent practice without institutional reinforcement.

Interpreted through Benner's competence framework (Benner 1982), this profile implies that many staff may still be developing specialised critical care expertise and therefore benefit from structured, repeated competency support rather than reliance on experiential learning alone. The very low proportion with formal critical care training (1.8%) strengthens the case for accessible in‐service education and locally feasible competency development pathways.

The workshop was associated with a statistically significant and clinically meaningful improvement in VAP‐prevention knowledge (pre‐test M = 3.63, SD = 1.57; post‐test M = 5.29, SD = 1.57; t(111) = −5.61, *p* < 0.001; *d* = 1.06), consistent with prior educational evaluations in critical care nursing (Benner 1982; Gundo et al. 2021). The strongest item‐level gains occurred in domains that typically require both conceptual understanding and routine decision‐making (e.g., humidifier change frequency, suction system selection, patient positioning and awareness of subglottic secretion drainage). These patterns suggest that a focused workshop can target common knowledge gaps that directly shape bedside choices; however, the extent to which improved knowledge translates into sustained bundle adherence depends on the feasibility of implementing those practices in the local context. The parallel non‐parametric result (*U* = 748.5, *p* < 0.001) provides reassurance that the observed change is robust despite departures from normality.

Knowledge‐based competence categories also shifted substantially after training: 64.3% were classified as high knowledge‐based competence post‐test compared with 24.6% pre‐test and the low knowledge‐based competence category fell to 1.8% (*χ* ^2^(2) = 22.43, *p* < 0.001). From a Bandura self‐efficacy perspective (Bandura and Adams 1977), these shifts are compatible with increased perceived capability to undertake VAP‐prevention behaviours following a mastery‐oriented learning experience. Importantly, 33.9% remained in the moderate category, indicating that a single, largely didactic exposure may not be sufficient for all nurses to consolidate knowledge into reliable clinical judgement, particularly for practices that are complex, infrequent, or resource dependent. This finding supports supplementing workshops with brief simulation/bedside coaching and ongoing feedback to move staff from ‘knowing’ to ‘doing’ (Akhter et al. 2025; Koukourikos et al. 2021; Wang et al. 2023). The integration of quantitative and qualitative findings revealed that although structured training significantly improved nurses' knowledge, this did not consistently translate into practice due to systemic constraints. While the quantitative data demonstrated measurable knowledge‐based competence gains, the qualitative findings highlighted underlying barriers such as resource limitations, lack of protocols and workload pressures. This integrated insight underscores the complexity of translating knowledge into practice and highlights the need for organisational and system‐level interventions alongside educational strategies.

The regression analysis found that years of RN experience, duration of ICU service and ICU type did not predict post‐test knowledge‐based competence (*F*(3, 52) = 0.94, *p* = 0.428, *R* ^2^ = 0.052). Rather than indicating that experience is unimportant, this result may reflect the way knowledge‐based competence is produced and maintained in this setting: when supervision is limited, protocols are absent and supplies are inconsistent, experience may accumulate without consistent reinforcement of evidence‐based standards. In such contexts, individual learning is less likely to be translated into shared practice because opportunities for feedback, standardisation and accountability are constrained. This interpretation is supported by the qualitative accounts of individualised practice and limited supervisory oversight. The low explanatory power of the regression model suggests that post‐training knowledge‐based competence may be shaped by factors not measured in this study, including organisational climate, leadership support, safety culture, motivation, perceived self‐efficacy, interprofessional collaboration and access to supervisory feedback.

A key contribution of the explanatory sequential design is that it clarifies why large knowledge gains may still fail to yield consistent prevention practice. Nurses described prioritising feasible ‘core tasks’ (notably oral care and suctioning) while reporting that supplies and equipment shortages, unreliable power and procurement constraints made other bundle elements difficult to deliver as recommended. Organisational factors, particularly the lack of standardised VAP protocols, limited in‐service training culture and weak supervision, further reduced consistency and accountability. Taken together, the findings suggest that knowledge‐based competence in VAP prevention is not solely an individual attribute, but an organisational outcome shaped by infrastructure, supply chains and leadership processes (Al‐Tamimi et al. 2022; Gatell et al. 2012; Toulabi et al. 2020). For clinical nursing leaders, the most actionable levers are the introduction of locally feasible protocols/checklists, routine audit‐and‐feedback and dependable access to basic consumables, alongside education.

From a critical care perspective, inconsistent implementation of VAP prevention practices has direct implications for patient outcomes, including increased risk of ventilator‐associated complications, prolonged ICU stay and mortality. Therefore, addressing system‐level barriers is essential not only for improving nursing knowledge‐based competence but also for enhancing overall quality of critical care delivery.

Because participant responses were not linked across assessment points, the observed improvement should be interpreted as a group‐level change rather than evidence of within‐individual improvement. Therefore, the findings support the potential educational value of the intervention but do not permit strong causal claims about individual knowledge‐based competence gains.

Overall, the study adds practice‐relevant evidence that a brief training workshop can substantially improve ICU nurses' VAP‐prevention knowledge in a resource‐limited setting, but that education alone is unlikely to close the gap between evidence and practice (Wang et al. 2023). A pragmatic way forward is to embed training within a small set of enabling supports: (1) a locally adapted VAP bundle protocol or checklist, (2) supportive supervision with audit‐and‐feedback and (3) reliable availability of essential supplies and equipment. Future studies should use linked (paired) designs where feasible and incorporate measures of bedside adherence and patient outcomes for example, VAP rates or ventilator days to determine whether combined ‘training plus system support’ strategies produce sustained clinical impact. The findings contribute to growing evidence that improving nurses' knowledge alone is insufficient to ensure effective infection prevention practices, particularly in resource‐limited settings where systemic constraints shape clinical behaviour.

From an implementation science perspective, the findings suggest that VAP prevention is influenced by interactions between individual capability, organisational readiness, available resources and implementation climate. Consistent with implementation‐oriented approaches such as the Consolidated Framework for Implementation Research, education should be accompanied by locally adapted protocols, audit‐and‐feedback systems, competency‐based mentoring, supportive supervision and reliable supply chains. These strategies may help bridge the gap between knowledge acquisition and sustained bedside adherence to VAP prevention bundles.

## 8 Limitations

Several limitations should be acknowledged. First, the single‐group quasi‐experimental design, while appropriate for this context, precludes causal inference as there is no concurrent control group. Second, the single‐site study limits generalisability to other settings with different resources and practices. Third, convenience sampling introduces selection bias. Fourth, participants were numbered sequentially across pre‐test and post‐test groups without a within‐subject linking variable, precluding paired analysis; an independent‐samples approach was therefore used, which is appropriate for the data structure but cannot account for within‐person change. Although the intervention targeted the same staff cohort, the absence of linked pre‐test and post‐test data limits the ability to assess within‐individual changes in knowledge‐based competence. Fifth, the study did not assess long‐term knowledge retention or impact on actual VAP incidence. Sixth, reliance on self‐reported barriers may have introduced social desirability bias. Seventh, the regression model explained little variance (*R* ^2^ = 0.052), suggesting that psychosocial, supervisory and organisational variables not measured in this study may be important predictors of post‐training knowledge‐based competence. Future research should employ paired or longitudinal designs with linked participant IDs and incorporate a broader range of determinants.

The quantitative component measured changes in knowledge and knowledge‐based competence but did not capture the contextual factors influencing practice. Conversely, the qualitative component provided in‐depth insights into these contextual barriers but was based on a small sample and may not be generalisable. Additionally, the inability to link individual pre‐test and post‐test responses limited the capacity to assess within‐person change, which the qualitative findings partially addressed by providing explanatory context. Thus, while each method had inherent limitations, their combination strengthened the overall interpretation of findings. The findings reflect group‐level changes and should not be interpreted as individual‐level improvements. The outcome measure primarily assessed knowledge‐based competence rather than observed bedside performance, VAP bundle adherence, or patient outcomes. The short interval between training and post‐test may have captured immediate knowledge gain rather than sustained retention. In addition, participants' awareness of being studied may have introduced a Hawthorne effect, particularly in self‐reported qualitative accounts of practice.

The internal consistency of the adapted VAP knowledge questionnaire was low (KR‐20 = 0.32 pre‐test; 0.42 post‐test), indicating that the eight items do not function as a unidimensional scale. The composite score should therefore be interpreted as a broad knowledge index rather than a precise psychometric measure. Future studies should consider developing and validating a locally adapted VAP knowledge instrument with stronger psychometric properties.

## 9 Recommendations

The findings of this study carry practical implications for multiple stakeholders involved in ICU nursing practice, education and policy in resource‐limited settings. While structured training demonstrably improves knowledge‐based competence, the qualitative evidence makes clear that sustained improvement in VAP prevention requires coordinated action beyond individual education. The following recommendations are organised by stakeholder to guide targeted and context‐sensitive responses.

### 9.1 ICU Nurses

- Apply evidence‐based VAP prevention bundles consistently, prioritising patient positioning, oral care and appropriate suctioning technique.
- Engage proactively in available in‐service training and seek out self‐directed learning opportunities to strengthen specialised critical care knowledge‐based competence.

### 9.2 Nurse Managers and Charge Nurses

- Develop and implement locally feasible written VAP prevention protocols and bedside checklists with regular audit‐and‐feedback to support consistent and accountable practice.
- Strengthen clinical supervision and create structured feedback mechanisms to reinforce evidence‐based practice and enable progression from knowledge‐based competence to bedside performance.

### 9.3 Hospital Administrators

- Prioritise procurement of essential consumables and equipment required for infection prevention, including suction catheters, closed‐system circuits and personal protective equipment.
- Address staffing and workload constraints that limit nurses' capacity to carry out preventive care, particularly oral hygiene and patient positioning.

### 9.4 Policymakers

- Invest in reliable power infrastructure, functional laboratory services and financial protection mechanisms that enable consistent infection prevention in critically ill patients.
- Develop national critical care nursing competency frameworks that include VAP prevention standards for resource‐limited ICU settings.

### 9.5 Researchers

- Conduct longitudinal or paired‐ID studies to assess retention of VAP‐prevention knowledge‐based competence, bedside bundle adherence and patient outcomes including VAP rates and ventilator days following combined training‐plus‐system‐support interventions.
- Evaluate the effectiveness of simulation‐based and observation‐assessed competency programmes in low‐resource ICU settings.

### 9.6 Clinical Implications for ICU Nursing Practice

The findings carry several direct implications for nursing practice in resource‐limited ICUs. First, education remains a necessary and impactful component of knowledge‐based competence development, as demonstrated by the large effect size achieved with a single brief workshop. Nurses and unit educators should advocate for regular, context‐adapted training that targets specific knowledge gaps identified in local practice. Second, knowledge alone is insufficient. Nurse managers and charge nurses play a critical role in translating education into practice through the introduction of written VAP prevention protocols or bedside checklists, regular audit‐and‐feedback cycles and structured supervisory rounds. Third, hospital administrators and procurement officers must ensure reliable access to essential consumables including suction catheters, face masks and ventilator circuits as equipment shortages directly undermine the application of even well‐learned prevention strategies. Fourth, at the health system level, investment in stable power infrastructure and functional laboratory services is a prerequisite for effective infection prevention in critically ill patients. Finally, for clinical nursing education, these findings reinforce the need to develop and evaluate competency‐oriented educational pathways incorporating simulation and observed performance assessment.

## 10 Conclusion

Structured training was associated with substantial improvements in ICU nurses' knowledge‐based competence in ventilator‐associated pneumonia prevention, accompanied by a large effect size and a marked increase in the proportion of nurses achieving high competence levels. However, persistent organisational and resource constraints limited the translation of knowledge into consistent clinical practice. Because pre‐test and post‐test responses were not linked, the findings should be interpreted as group‐level rather than within‐individual improvements. To achieve sustainable gains in resource‐limited ICUs, educational interventions should be integrated with quality improvement strategies, including locally feasible protocols, supportive supervision, audit‐and‐feedback mechanisms and reliable access to essential supplies and equipment.

## Author Contributions

**Yakubu H. Yakubu:** conceptualization, methodology, formal analysis, investigation, supervision, writing – original draft, writing – review and editing. **Muniru Mohammed Saani:** conceptualization, methodology, investigation, project administration, data curation, writing – review and editing.

## Funding

The authors have nothing to report.

## Disclosure

This article is derived from a postgraduate thesis submitted to the Ghana College of Nurses and Midwives in partial fulfilment of the requirements for membership of the Faculty of Critical Care Nursing (Muniru Mohammed Saani, 2023, unpublished thesis).

## Ethics Statement

Ethical clearance was obtained from the Navrongo Health Research Centre Institutional Review Board (NHRCIRB519, approval date: 15th May 2023). Institutional permission was granted by the Tamale Teaching Hospital Chief Executive Officer (TTH/R&D/SR/147, dated 8th June 2023). All participants provided written informed consent. Participation was voluntary and confidentiality was maintained throughout. This study was conducted in accordance with the Declaration of Helsinki.

## Conflicts of Interest

The authors declare no conflicts of interest.

## Supporting information


**Data S1:** Supporting Information.

**Data S2:** Supporting Information.

**File S1:** Interview Guide.

**Table S2:** Good Reporting of a Mixed‐Methods Study (GRAMMS) checklist.

**File S3:** Thematic Framework of Qualitative Findings.
**Table S3:** Major Themes and Subthemes from Qualitative Analysis of ICU Nurses' Experiences in VAP Prevention.

## Data Availability

The data that support the findings of this study are available from the corresponding author upon reasonable request.
