# Supplementary material for: Effect of Structured Training on ICU Nurses' Knowledge‐Based Competence in Ventilator‐Associated Pneumonia Prevention in a Resource‐Limited Setting: An Explanatory Sequential Mixed‐Methods Study
Source: Nurs Open. 2026 Jun 23;13(7):e70662. doi: 10.1002/nop2.70662 (PMC13291207; doi:10.1002/nop2.70662)
Supplement: Supplementary file 3 — File S1: Interview Guide. [file NOP2-13-e70662-s005.docx]

**Supplementary File 1: Interview Guide**

**Title:**
**Supplementary File 1. Semi-Structured Interview Guide for ICU Nurses on Ventilator-Associated Pneumonia (VAP) Prevention**

**Purpose of the Interview**

This semi-structured interview guide was developed to explore ICU nurses’ experiences, perceptions, and challenges related to ventilator-associated pneumonia (VAP) prevention. The guide was informed by the quantitative findings of the study and designed to provide explanatory insights into nurses’ competence, clinical practices, and contextual barriers within a resource-limited ICU setting.

**Section 1: Knowledge and Routine Clinical Practice**

1. **Can you describe how you usually prevent ventilator-associated pneumonia in your daily ICU practice?**
   *Probe:* What specific steps or procedures do you routinely follow?
2. **Which VAP prevention practices do you feel most confident performing, and why?**
   *Probe:* Are there practices you find easier or more effective than others?

**Section 2: Learning and Competence Development**

1. **How did you learn about VAP prevention?**
   *Probe:* Through training, clinical experience, colleagues, or self-learning?
2. **Do you think your current knowledge and skills are sufficient to effectively prevent VAP? Why or why not?**
   *Probe:* Are there areas where you feel less confident?

**Section 3: Resource and Environmental Factors**

1. **What challenges do you face in your work environment when trying to prevent VAP?**
   *Probe:* Equipment availability, supplies, infrastructure (e.g., electricity), or other factors?

**Section 4: Organisational and Training Factors**

1. **Are there any guidelines or protocols in your unit for preventing VAP? How do they influence your practice?**
   *Probe:* Are these protocols clear, available, and consistently followed?
2. **How do training and in-service education in your facility support your ability to prevent VAP?**
   *Probe:* What makes training helpful or not helpful?

**Section 5: Supervision, Workload, and System Support**

1. **How do supervision, workload, and staffing affect your ability to consistently apply VAP prevention practices?**
   *Probe:* How does patient load or support from senior staff influence your work?

**Closing Question**

1. **Is there anything else you would like to share about your experience with VAP prevention in your unit?**
