# Supplementary material for: Effect of Structured Training on ICU Nurses' Knowledge‐Based Competence in Ventilator‐Associated Pneumonia Prevention in a Resource‐Limited Setting: An Explanatory Sequential Mixed‐Methods Study
Source: Nurs Open. 2026 Jun 23;13(7):e70662. doi: 10.1002/nop2.70662 (PMC13291207; doi:10.1002/nop2.70662)
Supplement: Supplementary file 4 — Table S2: Good Reporting of a Mixed‐Methods Study (GRAMMS) checklist. [file NOP2-13-e70662-s004.docx]

Supplemental File Table S2: **Good Reporting of a Mixed-Methods Study (GRAMMS) checklist**

| Item | Section. Page |
| --- | --- |
| (1) Describe the justification for using a mixed methods approach to the research question | [Method] Design  Page 4 |
| (2) Describe the design in terms of purpose, priority and sequence of methods | [Method] Design  Page 4-5 |
| (3) Describe each method in terms of sampling, data collection and analysis | [Method]  Page 5-6 |
| (4) Describe where integration has occurred, how it has occurred and who has participated in it | [Method]  Page 4 and 14-18 |
| (5) Describe any limitation of one method associated with the present of the other method | [Discussion]  Strengths and Limitations  Page 18- 21 |
| (6) Describe any insights gained from mixing or integrating methods | [Results] Page 9 |

O’Cathain, A., Murphy, E., & Nicholl, J. (2008). The quality of mixed methods studies in health services research. *Journal of Health Services Research and Policy*, *13*(2), 92–98. https://doi.org/[10.1258/jhsrp.2007.007074](https://doi.org/10.1258/jhsrp.2007.007074)
