# Supplementary material for: Effect of Structured Training on ICU Nurses' Knowledge‐Based Competence in Ventilator‐Associated Pneumonia Prevention in a Resource‐Limited Setting: An Explanatory Sequential Mixed‐Methods Study
Source: Nurs Open. 2026 Jun 23;13(7):e70662. doi: 10.1002/nop2.70662 (PMC13291207; doi:10.1002/nop2.70662)
Supplement: Supplementary file 5 — File S3: Thematic Framework of Qualitative Findings. Table S3: Major Themes and Subthemes from Qualitative Analysis of ICU Nurses' Experiences in VAP Prevention. [file NOP2-13-e70662-s003.docx]

| **Major Theme** | **Subtheme** | **Description** |
| --- | --- | --- |
| 1. Practical Knowledge and Routine VAP Prevention Practices | Emphasis on basic, feasible preventive practices | Nurses prioritised routine, task-based interventions such as mouth care, suctioning, and ventilator circuit maintenance, reflecting a pragmatic approach shaped by feasibility within resource constraints. |
|  | Infection prevention knowledge acquired through experience | Knowledge of VAP prevention was largely derived from clinical experience and self-directed learning, with limited reliance on formal training or institutional support. |
| 2. Resource and Infrastructure Constraints | Limited availability of equipment and consumables | Shortages of essential supplies, including suction catheters and personal protective equipment, constrained adherence to infection prevention protocols and led to improvisation in practice. |
|  | Unstable power supply and system-level failures | Frequent power interruptions disrupted clinical procedures and compromised the safe and timely implementation of VAP prevention measures. |
| 3. Organisational and Training-Related Barriers | Absence of standardised VAP prevention protocols | Lack of formal, evidence-based protocols resulted in reliance on individual knowledge and inconsistent implementation of VAP prevention practices. |
|  | Low engagement in in-service training | In-service training was perceived as insufficient and undervalued, with poor participation influenced by staff attitudes, workload, and organisational limitations. |
| 4. Supervision, Workload, and Systemic Support Challenges | Inadequate clinical supervision and feedback | Limited supervisory oversight and absence of accountability mechanisms reduced opportunities for reinforcement, feedback, and standardisation of practice. |
|  | High workload and staffing pressures | High patient-to-nurse ratios and workload demands constrained nurses’ ability to consistently implement preventive care practices. |
|  | Delayed diagnostic feedback and family financial constraints | Delays in laboratory results and reliance on family financial support hindered timely clinical decision-making and continuity of care. |

**Supplementary file 3: Thematic Framework of Qualitative Findings**

Table S3. Major Themes and Subthemes from Qualitative Analysis of ICU Nurses’ Experiences in VAP Prevention
